# Comparative kinetic analysis on thermal degradation of some cephalosporins using TG and DSC data

**DOI:** 10.1186/1752-153X-7-70

**Published:** 2013-04-17

**Authors:** Adriana Fuliaş, Gabriela Vlase, Titus Vlase, Codruţa Soica, Alina Heghes, Marius Craina, Alina Heghes, Ionuţ Ledeti

**Affiliations:** 1 University of Medicine and Pharmacy “Victor Babeş”, Faculty of Pharmacy, Eftimie Murgu Square 2, Timişoara, RO-300041, ROMÂNIA; 2 West University of Timişoara, Research Centre for Thermal Analysis in Environmental Problems, Pestalozzi Street 16, Timişoara, RO-300115, ROMANIA; 3 University of Medicine and Pharmacy “Victor Babeş”, Faculty of Medicine, Eftimie Murgu Square 2, Timişoara, RO-300041, ROMÂNIA

**Keywords:** Cephalosporin, Kinetic parameters, Non-isothermal conditions, TG/DTG, DSC, NPK method

## Abstract

**Background:**

The thermal decomposition of cephalexine, cefadroxil and cefoperazone under non-isothermal conditions using the TG, respectively DSC methods, was studied. In case of TG, a hyphenated technique, including EGA, was used.

**Results:**

The kinetic analysis was performed using the TG and DSC data in air for the first step of cephalosporin’s decomposition at four heating rates. The both TG and DSC data were processed according to an appropriate strategy to the following kinetic methods: Kissinger-Akahira-Sunose, Friedman, and NPK, in order to obtain realistic kinetic parameters, even if the decomposition process is a complex one.

The EGA data offer some valuable indications about a possible decomposition mechanism. The obtained data indicate a rather good agreement between the activation energy’s values obtained by different methods, whereas the EGA data and the chemical structures give a possible explanation of the observed differences on the thermal stability. A complete kinetic analysis needs a data processing strategy using two or more methods, but the kinetic methods must also be applied to the different types of experimental data (TG and DSC).

**Conclusion:**

The simultaneous use of DSC and TG data for the kinetic analysis coupled with evolved gas analysis (EGA) provided us a more complete picture of the degradation of the three cephalosporins. It was possible to estimate kinetic parameters by using three different kinetic methods and this allowed us to compare the *E*_*a*_ values obtained from different experimental data, TG and DSC. The thermodegradation being a complex process, the both differential and integral methods based on the single step hypothesis are inadequate for obtaining believable kinetic parameters. Only the modified NPK method allowed an objective separation of the temperature, respective conversion influence on the reaction rate and in the same time to ascertain the existence of two simultaneous steps.

## Background

In this article it was realized a study about the kinetic parameters features of three active subtances- monohydrate form of cephalexin, cefadroxil, and cefoperazone. The kinetic parameters were obtained both from thermogravimetrical data and from DSC data.

The three active subtances belong to the cephalosporin class and are characterized by a common skeleton of cephem type. On this basic structure, different groups (radicals) are grafted that induce different bio-pharmacological properties of the three studied compounds.

Cephalosporins are bactericidal and have the same mode of action as other beta-lactam antibiotics but are less susceptible to penicillinases. Cephalosporins disrupt the synthesis of the peptidoglycan layer of bacterial cell walls. Cephalosporins are indicated for the prophylaxis and treatment of infections caused by bacteria susceptible to this particular form of antibiotic. First-generation cephalosporins (cephalexin, cefadroxil) are predominantly active against *Gram-positive* bacteria, and successive generations have increased activity against *Gram-negative* bacteria (cefoperazone) [1-3].

**Figure 1:**
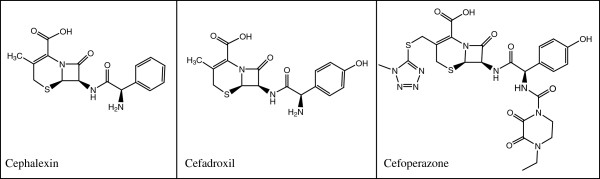
The chemical structures of the cephalosporin active substances.

Cephalosporin’s formulas are presented in Figure 1.

In order to be able to propose a methodology for the stability of these active substances, it is of paramount importance to understand their degradation mechanisms. The degradation process occurs by bond breaking, which generates smaller molecules.

Most techniques that are used to follow the reaction, either by analyzing gas-phase products [4,5] or by thermogravimetric analysis [6,7], will only detect the reaction when the products have become sufficiently small to evaporate into the gas phase.

A wide range of models have been developed, especially based on the analyses of weight loss curves obtained during thermal gravimetric analysis (TG kinetic models) and on the reaction mechanisms [7], for different materials [8-10]. However, the use of models based on partial information, like the one supplied by TG curves, cannot provide a clear view of the whole process since, in the initial stages, the degradation of substance proceeds without weight loss.

In our previous papers we provided the importance and utility of the kinetic analysis in estimation on the thermal behaviour of different pharmaceuticals [11-13].

## Methods

### Materials

The active substances (cephalexin, cefadroxil, cefoperazone) were obtained from Antibiotice Iaşi România.

The thermoanalytical curves TG/DTG/DTA/DSC for all the three active substances were drawn up in an air atmosphere and under dynamic conditions at different heating rates using Perkin-Elmer DIAMOND equipment, respectively a Netzsch differential scanning calorimeter, model DSC-204.

The DSC and TG data were recorded both under non-isothermal conditions. Samples of mass in the range of 3 to 7 mg were put into aluminium crucibles, at a heating rate, β, of 5, 7, 10 and 12°C·min^-1^ up to a temperature of 500°C.

The evolved gas analysis (EGA) was carried out by a coupled TG/FTIR technique, using a Perkin Elmer SPECTRUM 100 device with an IR gas chamber connected by a transfer line to the exit of the DIAMOND furnace. The air flow of 100 mL·min^-1^ and a heating rate of 20°C·min^-1^ were used. The FTIR spectra were processed by the Sadtler Gas Vapor Library.

## Results and discussion

Thermal behaviours of the three cephalosporin monohydrate active substances are characterized by the existence of two stages which can be identified both by the TG and DSC curves. The processes that follow are still numerous and consist in destroying the whole product from dihydrotiazinic cycle decomposition (cephem structure).

**Figure 2:**
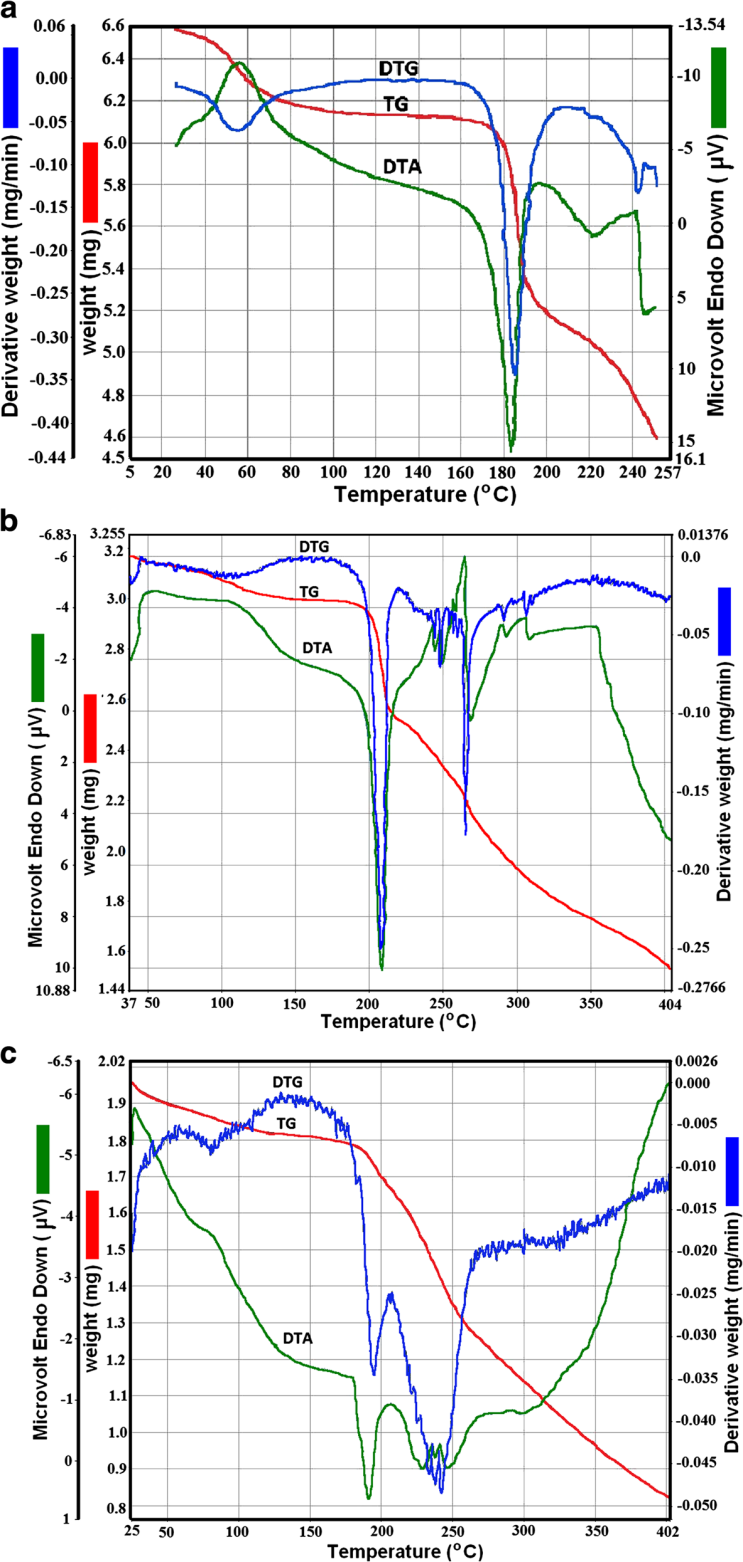
**The thermoanalytical curves TG/DTG/DTA obtained in air at β=5°C·min**^**-1 **^**for the monohydrate active substances (a- cephalexin; b- cefadroxil; c- cefoperazone).**

**Figure 3:**
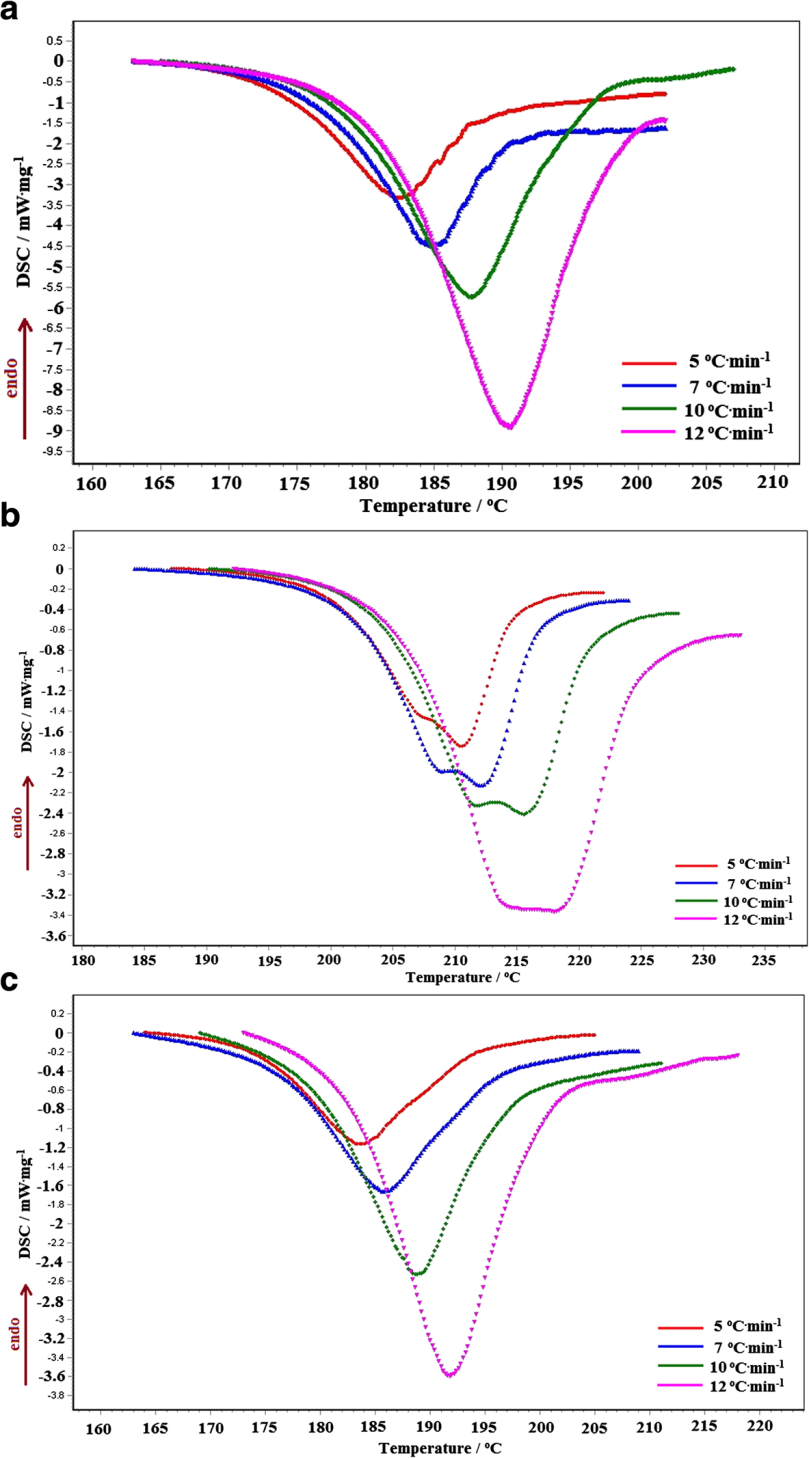
The DSC curves obtained in air at four different heating rates for the monohydrate active substances (a- cephalexin; b- cefadroxil; c- cefoperazone).

The results of TG/DTG/DTA obtained for the active substances during heating at β=5°C·min^-1^ in air atmosphere are presented in Figure 2, whereas the DSC data are presented in Figure 3.

The kinetic analysis is performed for the second decomposition process, the process which took place in the approximately temperature range 175-225°C. The first decomposition process corresponds to removal of water molecule from the composition of the hydrate. The dehydration of the sample with loss of one water molecule took place in the range 50-150°C.

The second process involves the destruction of cephalosporin molecule and for this reason it was selected for the kinetic analysis. What happens after this step does not present any interest for the characterization of the thermal behaviour.

To perform the kinetic analysis of the experimental data such as TG and DSC, there were used two isoconversional methods, a differential one and an integral one, respectively a third method elaborated by Sempere et al. [14,15] and modified and developed by Vlase et al. [16-18], the non-parametric kinetics method.

The isoconversional methods which evaluate the activation energy are advantageous as they do not require the knowledge of the analytical form of conversion function and, on the other hand, they give the possibility to evidence the change of the activation energy with the conversion degree.

a) Friedman method

Friedman’s isoconversional method [19] is based on the equation:

(1) $$\ln\left(\beta \frac{\mathrm{d\alpha}}{\mathrm{dT}}\right)=\ln\left[A.f\left(\alpha \right)\right]-\frac{E}{R.T}$$

The necessary data for graphical representation $\ln\left(\beta \frac{\mathrm{d\alpha}}{\mathrm{dT}}\right)$*vs.* (1/T) were taken from the chart of the reaction rate *vs.* temperature, under non-isothermal conditions. It was obtained the Friedman straight lines family, from the slope of those lines being calculated the activation energy according to Eq. (1).

The activation energy’s values corresponding of the second decomposition process which appears in all of three cephalosporin’s thermal behaviour, the most important one, according to the degree of conversion are presented in Table 1.

The data from Table 1 indicate that the values of *E*_*a*_ are in the following order:

Cefoperazone < Cephalexin < Cefadroxil

respectively *E*_*a*_ values somewhat higher by processing the DSC data in comparison with that from the TG ones.

It is noted a significant variation of the activation energy depending on the conversion degrees. This fact indicates that the decomposition process is complex, respectively the existence of more than one process. In this case it is necessary to use other kinetic methods of study, more developed in an attempt to determine and separate these processes, yet unknown as number.

b) Kissinger-Akahira-Sunose method [21]

This method is based on measuring the temperature corresponding to fixed values of the conversion degrees (α) for experiments at different heating rates β. Using TG respectively DSC curves recorded at various heating rates, one can plot $\ln\frac{\beta }{T^{2}}$*vs.* 1/T. According to Eq. 2, from the slope of the obtained straight line, the activation energy can be calculated. If it noticed a change of the *E*_*a*_ values function of α, the result can be interpreted as a multistage reaction mechanism.

This method is based on measuring the temperature corresponding to fixed values of the conversion degrees (α) for experiments at different heating rates β. Using TG respectively DSC curves recorded at various heating rates, one can plot $\ln\frac{\beta }{T^{2}}$*vs.* 1/T. According to Eq. 2, from the slope of the obtained straight line, the activation energy can be calculated. If it noticed a change of the *E*_*a*_ values function of α, the result can be interpreted as a multistage reaction mechanism.

(2) $$\ln\frac{\beta }{T^{2}}=\ln\frac{A.R}{E.g\left(\alpha \right)}-\frac{E}{R.T}$$

By inspecting the data in Table 2, there have been done the same comments as those made by Friedman’s method, i.e.:

a significant variation of *E*_*a*_ vs. α;

a general higher *E*_*a*_ values from DSC in comparison with that from TG;

the *E*_*a*_ values varies in the following order: Cefoperazone < Cephalexin < Cefadroxil

c) Non-parametric kinetics method (NPK)

**Table 1:** Activation energy’s values obtained by Friedman isoconversional method for the three analyzed cephalosporins from DSC data (a) respectively TG data (b)

***E***_***a ***_**kJ mol**^**-1**^	**Conversion degree α**									${\bar{E}}_{a}$ **kJ mol**^**-1**^
	**0.1**	**0.2**	**0.3**	**0.4**	**0.5**	**0.6**	**0.7**	**0.8**	**0.9**	
**Cefadroxil (a)**[20]	510.3	475.9	468.3	399.5	355.7	331.1	259.1	245.8	194.9	360.1
	±1.0	±0.5	±1.4	±2.8	±12.8	±3.6	±7.5	±9.9	±10.8	±37.4
**Cephalexin (a)** [11	257.7	249.9	256.8	251.8	255.8	243.5	266.2	284.5	295.1	262.4
	±2.3	±4.0	±5.9	±2.5	±3.8	±7.0	±12.5	±5.7	±9.8	±5.6
**Cefoperazone (a)**	147.0	164.4	189.6	202.8	196.5	186.1	192.6	204.3	269.8	194.8
	±3.3	±2.8	±4.1	±1.7	±2.9	±6.2	±4.0	±9.1	±8.7	±11.2
**Cefadroxil (b)**	373.6	432.6	403.3	381.5	306.8	300.9	211.3	159.2	118.4	298.6
	±5.2	±3.9	±2.0	±1.3	±1.5	±13.2	±6.9	±13.2	±2.7	±37.5
**Cephalexin (b)**	272.9	257.2	247.1	253.5	246	232.5	224.7	231.9	209.7	241.7
	±3.2	±7.4	±2.4	±13.6	±11.2	±33.6	±8.4	±9.6	±8.8	±6.3
**Cefoperazone (b)**	168.8	195.5	192.1	165.3	68.1	39.9	61.1	131.0	271.7	143.7
	±3.8	±4.1	±6.4	±3.9	±2.2	±8.6	±8.6	±3.3	±15.0	±25.3

**Table 2:** The activation energy values obtained by the KAS method for the three analyzed active substances utilised DSC data (a) respectively TG data (b)

***E***_***a ***_**kJ mol**^**-1**^	**Conversion degree α**									${\bar{E}}_{a}$ **kJ mol**^**-1**^
	**0.1**	**0.2**	**0.3**	**0.4**	**0.5**	**0.6**	**0.7**	**0.8**	**0.9**	
**Cefadroxil**	283.1	283.3	283.2	287.1	284.1	286.5	283.0	285.8	290.8	285.2
**(a)**[20]	±5.5	±8.9	±3.3	±3.4	±7.4	±1.5	±4.5	±1.1	±2.2	±0.9
**Cephalexin**	236.7	247.1	249.6	250.2	251.4	254.4	261.4	264.6	270.8	254.0
**(a)**[11]	±17.3	±5.7	±2.3	±1.5	±0.3	±0.9	±1.8	±12.9	±2.4	±3.4
**Cefoperazone**	171.8	192.5	188.0	189.6	189.0	190.7	173.2	182.6	179.8	184.1
**(a)**	±5.5	±3.8	±3.7	±3.8	±3.8	±3.9	±2.4	±1.3	±0.6	±2.6
**Cefadroxil**	389.2	379.9	366.7	370.9	363.2	354.2	340.7	338.6	336.2	359.9
**(b)**	±2.7	±3.4	±1.8	±1.3	±1.8	±8.5	±4.4	±7.8	±4.4	±6.3
**Cephalexin**	242.7	240.2	231.0	224.7	213.6	239.9	240.4	243.0	257.1	236.9
**(b)**	±1.2	±2.2	±7.6	±2.3	±4.1	±4.8	±18.5	±9.0	±19.3	±4.2
**Cefoperazone**	221.3	186.4	182.7	178.5	165.8	170.2	148.6	140.8	141.9	170.7
**(b)**	±8.6	±4.6	±3.9	±4.0	±2.6	±7.1	±3.3	±5.1	±1.9	±8.5

The non-parametric kinetics (NPK) represents a special approach for processing the kinetic data. The method introduces a new point of view in kinetic analysis. It is also based on the single-step kinetics approximation, so that the basic relationship for the analysis of kinetic data represents Eq. 3:

(3) $$\frac{\mathrm{d\alpha}}{\mathrm{dT}}=k\left(T\right).f\left(\alpha \right)$$

The experimental values of reaction rates are arranged in a matrix which is expressed as a product of two vectors containing information on k(T) and f(α). The most important feature of the method is that it enables to decouple the vectors related to the temperature and conversion functions without the need of any assumptions about their functionality. Validity of Eq. 3 is the only assumption made in the development of NPK method.

**Figure 4:**
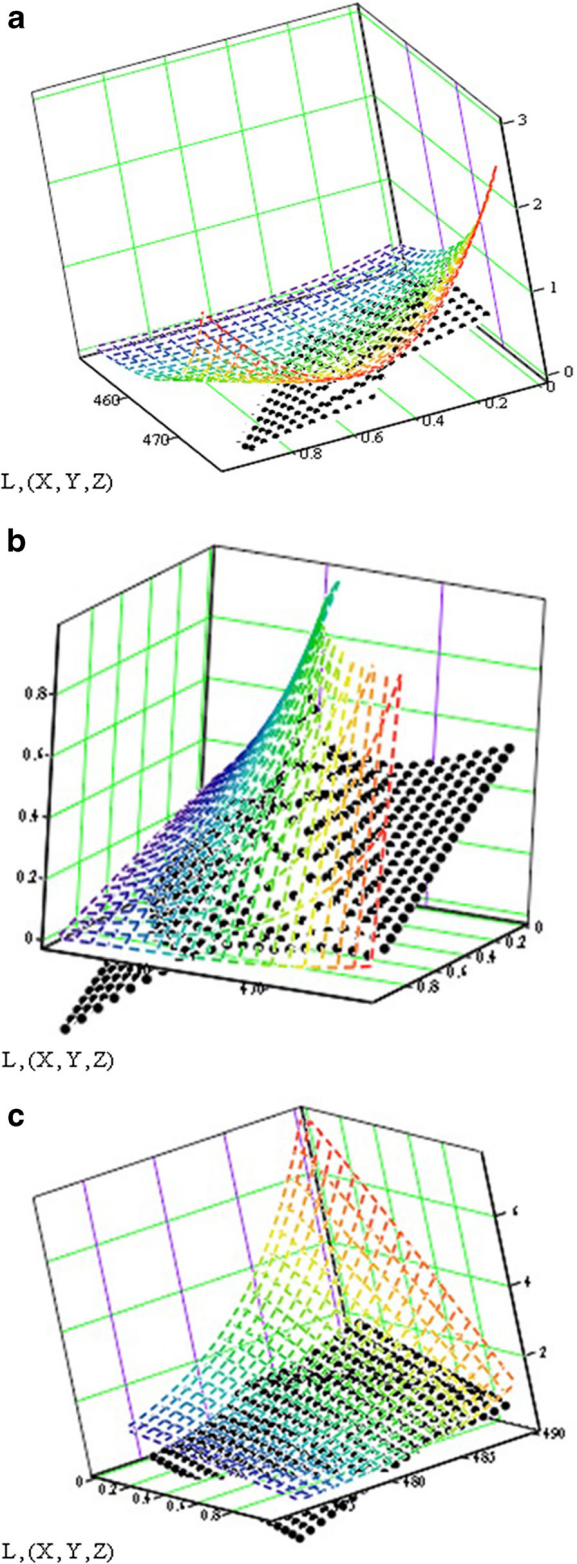
The reaction rate surfaces in the three-dimensional space with the coordinates (β·dα/dT; α; T) for the analysed cephalosporins.

The experimental points obtained at different heating rates are represented in a 3D system and interpolated as a continuous reaction rate surface (see Figure 4).

This surface is discretizated into a square matrix M which is decomposed, using the singular value decomposition algorithm [22], into the product of three matrixes:

(4) $$M=U\left(\mathrm{diag}S\right)V^{T}$$

The results of NPK analysis are systematized in Table 3. These data were obtained by analyzing the vector u (the first column of U) in respect of a kinetic model suggested by Sestak and Berggren [23]:

(5) $$f\left(\alpha \right)=\alpha^{m}.{\left(1-\alpha \right)}^{n}$$

respectively the vector v (the first column of V) for an Arrhenius type temperature dependence.

The value of the explained variance, λ, in Table 3, expresses the contribution of two simultaneous steps to the whole thermodegradation process, so that Σ λ_i_=100%. If λ<10%, we consider that the discussed step can be neglected. From this point of view, there are significant differences in the thermal behaviour of the three samples: only cefadroxil seems to be thermodegradated into a single step, whereas cephalexin and especially cefoperazone are degradated by a process with two significant parallel steps.

**Table 3:** Kinetic analysis for the three active substances, the NPK method

	**Process**	**λ (%)**	**E (kJ mol**^**-1**^**)**	**A (min**^**-1**^**)**	**n**	**m**	**Sestak-Bergrren Eq**	**∑λ**·**E (kJ mol**^**-1**^**)**
***Cefadroxil***	1	95.4	258.6±7.1	1.33·10^27^	1	-	(1-α)	264.5±9.8
(DSC data) [20]	2	4.6	385.4±83.8	1.07·10^37^	-	1/3	α^1/3^	
***Cephalexin***	1	77.2	220.2±9.7	8.04·10^23^	1	-	(1-α)	248.0±18.2
(DSC data) [11]	2	18.9	412.4±162.2	3.40·10^45^	5/3	1	α^5/3^ · (1-α)	
***Cefoperazone***	1	86.7	170.2±17.4	2.70·10^18^	2	1	α · (1-α)^2^	179.9±14.9
(DSC data)	2	13.3	243.8±17.6	7.18·10^26^	2	1	α · (1-α)^2^	
***Cefadroxil***	1	93.9	226.7±8.3	4.95·10^23^	2	1	α · (1-α)^2^	231.9±14.1
(TG data)	2	4.5	428.1±40.3	7.88·10^44^	-	0.1	α^0.1^	
***Cephalexin***	1	84.0	181.2±13.2	7.37·10^19^	1	-	(1-α)	220.6±13.2
(TG data)	2	16.0	411.9±75.4	5.10·10^45^	0.1	-	(1-α)^0.1^	
***Cefoperazone***	1	71.4	141.5±6.0	9.16·10^14^	3	-	(1-α)^3^	156.4±12.4
(TG data)	2	22.5	245.6±83.6	5.80·10^26^	-	3	α^3^	

Regarding the values of *E*_*a*_, by DSC data processing, higher values were obtained in comparison with that one from TG data. Probably, this is in connection with the different kinetic models by DSC, respectively TG data (see Table 3).

Concerning the mean values of *E*_*a*_ (∑*λ·E*_*a*_), the same rule was observed, i.e. Cefoperazone < Cephalexin < Cefadroxil.

In order to elucidate the basic reactions of thermal degradation of these active substances in oxidative atmosphere, it was realised a study on identification and tracing of evolving gaseous species from pure cephalosporins monohydrate using coupled FTIR spectrometric gas cell as detector connected to furnaces of thermal balances (TG-FTIR). The components of released gaseous mixtures have been monitored and identified mostly on the basis of their FTIR spectra.

Arguments for a rapid thermooxidation of the molecule were brought by EGA by identifying the substances which arise from both the destruction of radicals and the destruction dihydrothiazine cycle. It was identifying three stages of decomposition: dehydration process; degradation of dihydrothiazine ring, respectively further decomposition with releasing of benzene, *p*-cresol, phenol, azine and N-ethyl-formamide from the substituents [11,20,24].

Similarity of the EGA spectra is observed throughout the analysed process; the wave numbers are identical and they characterize the existence of the same compounds which result from total breakdown of the initial active substances.

**Figure 5:**
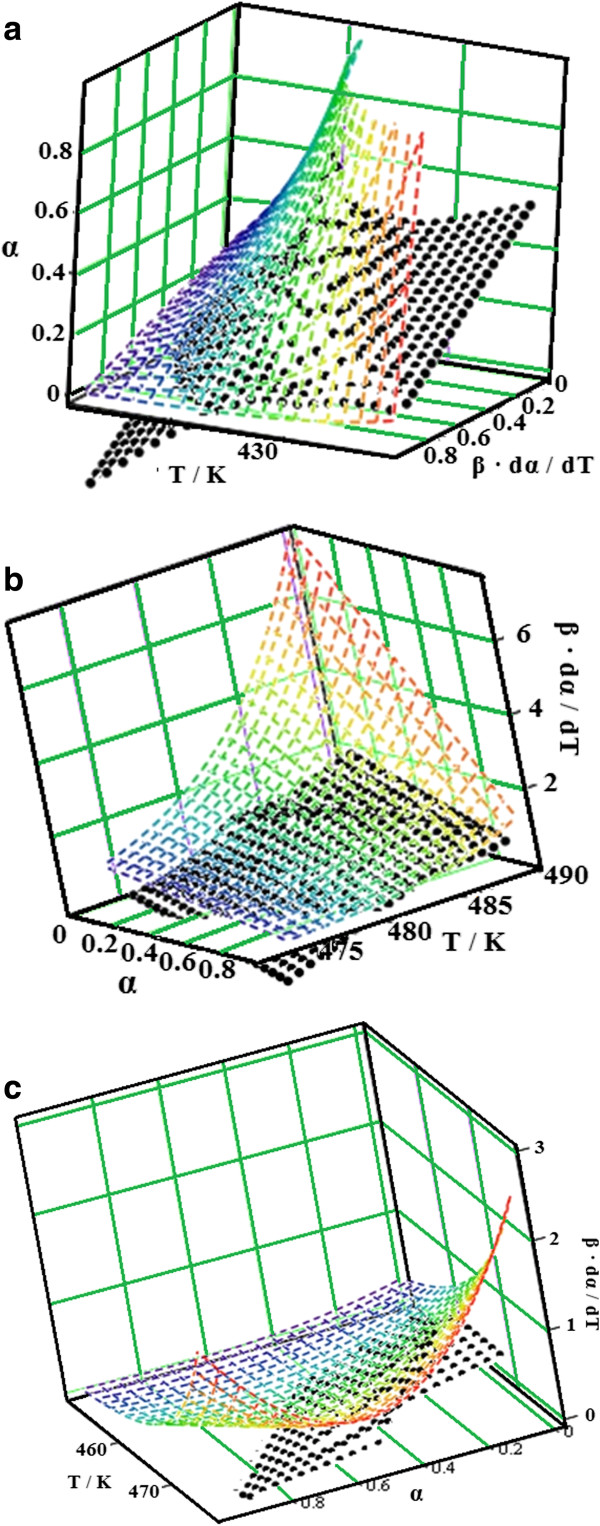
The connections between the structure of the three active substances and EGA results.

The compounds that can be identified from the EGA spectra are: water vapours (ν=3750-3500 and 1900–1300 cm^-1^); carbon dioxide (ν=2300-2250 and 750–600 cm^-1^); carbon sulphide (ν=750-700 cm^-1^); ethyl-formamide (ν=1680-1660 cm^-1^ - stretching vibration C=O from amide; 1560–1530 cm^-1^ - stretching vibration N-H, respectively C-N); azine (ν=1615-1565 cm^-1^ - stretching vibration of C=N bond from aromatic cycle); phenol (ν=3550-3500 cm^-1^ - stretching vibration of O-H phenolic bond). These data are systematized in Figure 5.

These products identified from the EGA spectra are according with the active substance’s skeleton and are demonstrating a significant destruction of dihydrothiazine ring (see carbon sulphide), but also of this ring’s substituents. The most complicated cefoperazone, with the azine ring and the sulphur bridge brings a low stability, whereas the cefadroxil, with the extended conjugation on the phenolic hydroxyl, exhibit the highest thermooxidative stability.

## Conclusions

The simultaneous use of DSC and TG data for the kinetic analysis coupled with evolved gas analysis (EGA) provided us a more complete picture of the degradation of the three cephalosporins.

It was possible to estimate kinetic parameters by using three different kinetic methods and this allowed us to compare the *E*_*a*_ values obtained from different experimental data, TG and DSC.

The thermodegradation being a complex process, the both differential and integral methods based on the single step hypothesis are inadequate for obtaining believable kinetic parameters. Only the modified NPK method allowed an objective separation of the temperature, respective conversion influence on the reaction rate and in the same time to ascertain the existence of two simultaneous steps. The observed order of *E*_*a*_’s values, i.e. Cefoperazone << Cephalexin < Cefadroxil is certainly in connection with the cephalosporin’s structure.

It was also shown that the use of TG data leads to lower values for activation energy, respectively for Sestak-Bergrren equation’s parameters. This aspect can be attributed to the fact that the TG data highlights only the mass loss. Compared with the TG data, the use of DSC data to determine the kinetic parameters allow the display of both chemical and physical processes which leads to relatively higher activation energy. Probably, this is also the reason of the observed differences by the conversion functions.

## Competing interests

The authors declare that they have no competing interests.

## Authors’ contributions

AF, GV and TV formulated the research idea and planned the experiment. IL, AH and CS had carried out the collection of data. AF and IL had processed results, had prepared the figures and tables and had finalized the manuscript. All authors participated in the article’s design and coordination and helped to draft the manuscript. All authors read and approved the final manuscript.

## References

[B1] Essack SY The development of beta-lactam antibiotics in response to the evolution of beta-lactamases Pham Res 2001 18 1391 1399 10.1023/A:1012272403776 11697463

[B2] James PA Reeves D Bacterial resistance to cephalosporins as a function of outer membrane permeability and access to their target J Chemother 1996 8 37 47 8738845

[B3] Crichlow GV Nukaga M Doppalapudi VR Buynak JD Knox JR Inhibition of class C beta-lactamases: Structure of a reaction intermediate with a cephem sulfone Biochem 2001 40 6233 9 10.1021/bi010131s 11371184

[B4] Serrano DP Aguado J Escola JM Garagorri E Performance of a continuous screw kiln reactor for the thermal and catalytic conversion of polyethylene - lubricating oil base mixtures Appl Catal B 2003 44 95 105 10.1016/S0926-3373(03)00024-9

[B5] Aguado J Serrano DP Escola JM Catalytic cracking of polyethylene over zeolite mordenite with enhanced textural properties Ind Eng Chem Res 2008 47 7982 7992 10.1021/ie800393w

[B6] Marcilla A Gomez-Siurana A Valdes F Catalytic cracking of low-density polyethylene over H-Beta and HZSM-5 zeolites: Influence of the external surface Kinetic model Polym Degrad Stab 2007 92 197 204 10.1016/j.polymdegradstab.2006.11.007

[B7] Wallis M Bhatia SK Kinetic study of the thermal degradation of high density polyethylene Polym Degrad Stab 2006 91 1476 1483 10.1016/j.polymdegradstab.2005.10.003

[B8] Kok MV Use of thermal equipment to evaluate crude oils Thermochim Acta 1993 214 315 324 10.1016/0040-6031(93)80068-L

[B9] Kok MV Coal pyrolysis: thermogravimetric study and kinetic analysis Energy Sources 2003 25 1007 1014

[B10] Peterson JD Vyazovkin S Wight CA Kinetics of the thermal and thermo-oxidative degradation of polystyrene, polyethylene and polypropylene Macromol Chem Phys 2001 202 775 784 10.1002/1521-3935(20010301)202:6<775::AID-MACP775>3.0.CO;2-G

[B11] Fuliaş A Vlase T Vlase G Doca N Thermal behaviour of cephalexin in different mixtures J Therm Anal Cal 2010 99 987 992 10.1007/s10973-010-0708-x

[B12] Vlase G Vlase T Doca N Thermal behavior of some phenitoine pharmaceuticals J Therm Anal Cal 2008 92 259 262 10.1007/s10973-007-8727-y

[B13] Vlase T Vlase G Doca M Doca N Specificity of decomposition of solids in non-isothermal conditions J Therm Anal Cal 2003 72 597 604 10.1023/A:1024537902405

[B14] Serra R Nomen R Sempere J The non-parametric kinetics. A new method for the kinetic study of thermoanalytical data J Therm Anal Cal 1998 52 933 943 10.1023/A:1010120203389

[B15] Serra R Sempere J Nomen R A new method for the kinetic study of thermoanalytical data: The non-parametric kinetics method Thermochim Acta 1998 316 37 45 10.1016/S0040-6031(98)00295-0

[B16] Vlase T Vlase G Doca N Ilia G Fuliaş A Coupled thermogravimetric-IR techniques and kinetic analysis by non-isothermal decomposition of Cd^2+^ and Co^2+^ vinyl-phosphonates J Therm Anal Cal 2009 97 467 472 10.1007/s10973-009-0219-9

[B17] Vlase T Vlase G Birta N Doca N Comparative results of kinetic data obtained with different methods for complex decomposition steps J Therm Anal Cal 2007 88 631 635 10.1007/s10973-006-8019-y

[B18] Vlase T Vlase G Doca N Bolcu C **Processing of non-isothermal TG data** Comparative kinetic analysis with NPK method J Therm Anal Cal 2005 80 59 64 10.1007/s10973-005-0613-x

[B19] Friedman HL Kinetics of thermal degradation of char-foaming plastics from thermogravimetry: application to a phenolic resin J Polymer Sci 1965 6C 183 95

[B20] Fuliaş A Doca N Vlase G Tiţa B Tiţa D Vlase T Thermal decomposition kinetics of cefadroxil Rev Roum Chim 2011 56 967 973

[B21] Akahira T Sunose T Joint convention of four electrical institutes. Research report (Chiba Institute of Technology Sci Technol 1971 16 22 31

[B22] Wall ME Berrar DP, Dubitzky W, Granzow M Singular value decomposition and principal component analysis A practical approach to microarray data analysis, Volume 9 2003 MA: Kluwer-Norwel 91 109

[B23] Sestak J Berggren G Study of the kinetics of the mechanism of solid-state reactions at increasing temperatures Thermochim Acta 1971 3 1 12 10.1016/0040-6031(71)85051-7

[B24] Fuliaş A Vlase G Vlase T Tiţa B Doca N Thermal study of cefoperazone monohydrate Rev Roum Chim 2010 55 481 6

